# Essential new-born care practices and associated factors among post natal mothers in Nekemte City, Western Ethiopia

**DOI:** 10.1371/journal.pone.0231354

**Published:** 2020-04-21

**Authors:** Bizuneh Wakuma Efa, Emebet Berhanie, Kalkidan Wondwossen Desta, Leta Hinkosa, Getahun Fetensa, Werku Etafa, Reta Tsegaye

**Affiliations:** 1 School of Nursing and Midwifery, Institute of Health Science, Wollega University, Nekemte, Ethiopia; 2 Department of Nursing and Midwifery, School of Allied Health Science, Addis Ababa University, Addis Ababa, Ethiopia; University of Gondar, ETHIOPIA

## Abstract

**Background:**

New-born survival is a prominent goal on the global health agenda and an important area of focus for programs seeking to ensure child survival. Geographically, neonatal deaths are most prevalent in Sub-Saharan Africa and southern Asia, accounting for 39% and 38% of all neonatal deaths respectively while Ethiopia in particular has 28% neonatal death. Promotion of essential new-born care practice is one of a cheap approach to improve health outcomes of new-born babies. Thus, this study was aimed to assess the magnitude of essential new-born care practices and associated factors among postnatal mothers in Nekemte city, Western Ethiopia.

**Methods:**

An institution-based cross-sectional study was conducted from February to March, 2017, in Nekemte city, East Wollega Zone. Data was collected from 417 randomly selected mothers who have less than six months infants by face to face interview in three public health institutions of Nekemte City, Ethiopia. Women who were not biological mother to the new-born were excluded from the study. The collected data were coded, cleaned and entered using Epi-Data version 3.1 and analysed using Statistical Package for Social Science (SPSS) version 21.0. Both bivariable and multivariable logistic regression analysis were computed to identify associated factors. The strength of association was measured by odds ratios with 95% confidence interval (CI) at a p-value of < 0.05 and finally obtained results were presented by using simple frequency tables, graphs, and charts.

**Results:**

The study revealed that the level of essential new-born care practice was 184(44.1%). The overall safe cord care practice of the respondents was 285 (68.3%) while the optimal thermal care practices and good neonatal feeding were 328 (78.7%) and 322 (77.2%) respectively. Having visit to Antenatal Care (ANC) [Adjusted Odds Ratio (AOR) = 4.38, 95% CI = (1.38, 13.94)], knowledge of essential new-born care [AOR = 4.58, 95% CI = (2.93, 7.16)], and counselled about essential new-born care [AOR = 2.32, 95% CI = (1.38, 3.91)] were factors significantly associated with good practices of essential new-born care.

**Conclusion:**

This study indicated that the level of essential new-born care practice was unsatisfactory in the study area. Promotion of essential new-born care through the provision of community awareness and provision of counselling on essential new-born care and neonatal danger signs to all pregnant women should be given emphasis.

## Background

New-born survival is a prominent goal on the global health agenda and an important area of focus for programs seeking to ensure child survival. By 2016, an estimated 2.6 million children died within the first 28 days after birth, or an average of about 7,000 every day. Currently Neonatal deaths accounts for two-thirds of all infant mortality and 46% of all under-five mortality, an increase from 41% in 2000. Among these, ninety-nine per cent of deaths occur in middle- and low-income countries. Though mortality at age 1–59 months declined by 62% between 1990 and 2016, the neonatal mortality rate (NMR) dropped by only 50%. Geographically, neonatal deaths are most prevalent in southern Asia and sub-Saharan Africa, accounting for 39% and 38% of all neonatal deaths, respectively in particular Ethiopia accounts for 29% of neonatal death. About three-fourths of deaths among new-borns occur in the first week of life, and 25–40% occurs in the first 24 hours [1–4]. Even though the causes of neonatal mortality are not well documented in Ethiopia, different studies report sepsis, asphyxia, birth injury, tetanus, preterm birth, congenital malformations as a cause [4].

Essential new-born care (ENC) is defined as a strategic approach planned to improve the health of new-borns through interventions before, during and after pregnancy, immediately after birth and during the postnatal period. Improving new-born survival begins with improving the health status of their mothers. It is a cost-effective intervention that improves both maternal and neonatal health as well as their nutritional status. Among care recommended to be given for all new-born baby, cord care, neonatal feeding and thermal care are practiced by mothers at home during the postnatal period [5–7].

Ethiopian Federal ministry of health has paid due attention to the expansion of quality high impact neonatal interventions by establishing basic new-born care units in health centres and neonatal intensive care units in hospitals [8]. It has also worked in increasing vaccination, Vitamin A, provision of insect treated nets (ITNs), family planning and water & sanitation to reduce under-five mortality in the last two decades [9]. Despite these efforts, neonatal death is still a noteworthy in Ethiopia. As the first 28 days of life of new-born are a most vulnerable time for an infant’s survival; particularly the first day, week and month of life, new-borns need a careful attention during this period to increase their survival rate and to improve their health outcomes through adhering to essential new-born care practice guidelines [5–7].

According to UN inter-agency group, neonatal mortality rate in Ethiopia is 28 deaths per 1000 live births in 2015 and hence with this regard Ethiopia has a huge homework to achieve Sustainable development goal (SDG) by 2030 which expect all countries aiming to reduce neonatal mortality to at least as low as 12 deaths per 1000 live births [10]. Essential new-born care is one good option to reduce neonatal mortality and morbidity. Despite this recommendation, there is inadequate adherence to it in middle- and low-income countries. The study conducted in various parts of Africa such as Uganda, Ghana and Cameroon reported that the prevalence of essential new-born care practice was low. For instance applying substances on cord stump and early bathing is a norm in Uganda [11–13]. Furthermore, studies done in different parts of Ethiopia also showed low essential new-born care practices [14, 15].

In Ethiopia, though limited numbers of studies have been conducted on essential new-born care practices, they were mainly confined to northern part of Ethiopia. According to these studies the essential new born care practices were varied from 13.7%-81.1% [14, 16–20]. As Ethiopia is multi ethnic and multicultural country, practice of essential new-born care and health seeking behaviour in general can be significantly varied across these settings. To the best of the researchers’ knowledge, little is known and there is no previous published work about practices of essential new-born care and factors affecting it in the western Ethiopia in general and study area in particular. Thus, this study was aimed to assess magnitude and associated factors of essential new-born care practices reported by postnatal mothers in Nekemte city, Western Ethiopia.

## Methods

### Study area and design

An institution-based cross-sectional study was conducted in Nekemte city public health facilities from February to March 2017. Nekemte city is located in the Western part of the Oromia region at 331 km away from country’s capital, Addis Ababa. The city has four public health institutions, namely Nekemte referral hospital, Wollega University referral hospital (started to function after data collection), Nekemte health centre, and Cheleleki health centres each giving health care service to the population of the town and nearby populations. According to Central Statistics Agency Branch in the town in 2016, the total population of the town is projected to be 97,289 among which young people in the town is estimated to be 37,796 (Male = 19,626, Female = 18,170) [21].

### Sample size and sampling procedures

A total of 417 mothers with an infant aged less than six months were included in the study. The sample size was computed using a single population proportion formula considering 52.1% proportion of timely breastfeeding initiation in four regions of Ethiopia [15], 95% confidence level, 5% marginal error, and 10% nonresponse rate, the final sample size was 422.

Systematic random sampling was used to select the study participants from the three public health facilities in Nekemte city namely; Nekemte referral hospital (NRH), Nekemte health centre (NHC) and Cheleleki health centre (CHC). The previous 3 months' clients flow to the three health facilities for Maternal and Child Health (MCH) service was reviewed from the registration book to estimate the expected number of mothers that visited the clinic in a month. Therefore, the average number of mothers visited the clinic in the previous three months back was 492, 243, and 135 for NRH, NHC, and CHC respectively.

The calculated sample size (422) was allocated to each health facility based on proportion to the population size of the 3 health facilities. Accordingly, proportional allocation to population size for each health facility was 239, 118 and 65 to Nekemte referral hospital, Nekemte health centre and Cheleleki health centre respectively. Sampling interval (k) was determined by dividing the total number of mothers expected to visit the MCH clinic of each health facility within a month by the number of sample size allocated to each health facility, thus sampling interval was approximately 2 for each health facility. Postnatal women paired to their infants who came to health facility during data collection period within six month of their delivery were included while those wo were not biological mother to the new-born were excluded from the study.

### Data collection tools and procedures

A semi-structured pretested interviewer-administered questionnaire was used. The tool was developed after exhaustively reviewing different relevant kinds of literature [14, 17–19]. The questionnaire comprises socioeconomic characteristics, information on health service utilization, mothers’ knowledge on new-born care and neonatal danger signs. The tool was prepared in the English version and it was translated into the regional working language, Afaan Oromo, and again translated back to the English language to check the consistency. The translated Afaan Oromo version questionnaire was used for data collection after pre-test in similar areas outside of the study site on 5% of the sample size before the actual data collection. The reliability of the questionnaire was checked by computing the cronbach’s alpha. Multicollinearity test was also done and reported by Variance inflation factor (VIF) which was 1.02.

Three data collectors and two supervisors were recruited from outside selected facilities. The purpose of the study was explained to them to minimize bias during data collection. The data collectors were trained for one day on basic principles of data collection, and how to gather information using interview. Furthermore, training on data completeness, cross-checking and corrective actions were given to the supervisors. The data were compiled, cleaned and entered at the end of each data collection day.

### Measurement

Essential new-born care: is a care provided to every new-born baby by postnatal mothers, which composed of neonatal feeding, cord care and thermal care.

Essential new-born care practice: The practice was reported ‘good’ for mothers who practiced three components (safe cord care, optimal thermal care, and good neonatal feeding) appropriately while the practice was reported ‘poor’ if at least one component was missed from three components [17–20].

Safe cord care: Defined as keeping the cord, clean and dry without application of any substance on the cord stump except medically indicated medications like chlorhexidine [17, 18].

Optimum thermal care: A new-born wrapped in clean and dry cloth and delay bathing a new-born delivery for 24 hours to prevent hypothermia [17, 18].

Neonatal feeding: Defined as initiating breastfeeding within the first one hour after birth, giving no pre-lacteal and feeding the child with colostrum [17, 18].

Knowledge of essential new-born care: Knowledge was ‘good’ for mothers who responded greater than 50% of knowledge related questions correctly whereas knowledge was ‘poor’ for mothers who responded less than or equal to 50% of knowledge related questions [17,18].

Knowledge of new-born danger sign- those mothers who identified at least 4 among the six listed danger signs categorized as good knowledge on neonatal danger sign and for those who mentioned less than four of danger signs were categorized as poor knowledge on neonatal danger sign.

### Data processing and analysis procedures

The collected data were entered into Epi data Version 3.1 and were analysed using SPSS version 20. Bivariate logistic regression analysis was used to see the significance of the association between dependent and independent variables. A P-Value of less than 0.05 was taken as statistically significant. Multivariable logistic regressions were used to identify associated factors & the strength of association was measured by odds ratios with 95% CI. Variables that had a significant association with the outcome variables in the crude analysis at p-value less than 0.2 were entered into the multivariable logistic regression model. In a Multivariable logistic regression model using adjusted odds ratio (AOR) independent predictors of new-born care practices among postpartum mothers were identified through controlling the confounding effects of other variables. Descriptive statistics were calculated and finally obtained results were presented by using simple frequency tables, graphs, and charts.

### Ethical considerations

Prior to data collection, ethical approval was obtained from ethical review committee of Addis Ababa University, school of Allied health sciences, department of Nursing and Midwifery with a reference number of 348/MSc/91/09. Official letter of permission was also obtained from the Oromia regional health bureau and Guto Gida district health bureau. Letter of cooperation from Guto Gida district health bureau was brought to the selected health facilities to get access to study participants. Respondents were told the aim of the study and informed written consent was obtained from the mothers before starting the interview.

## Results

### Socio-demographic characteristics of the participants

A total of 417 post-partum women were participated in the study yielding a response rate of 98.8%. The women’s mean age and interquartile range was 25 (22, 27). The majority of the respondents, 408 (97.8%) were married. Regarding their religion, above half of the respondents 254 (60.9%) were Protestant (Table 1).

**Table 1:** Socio-demographic characteristics of respondents, Nekemte city, Western Ethiopia, 2017.

Variables	Frequency	Percent
**Mothers Age**		
15–24	199	47.7
25–34	201	48.2
35–44	17	4.1
**Religion**		
Protestant	254	60.9
Orthodox	105	25.2
Muslim	53	12.7
Catholic	5	1.2
**Ethnic Groups**		
Oromo	374	89.7
Amhara	34	8.2
Others*	9	2.2
**Marital Status**		
Married	408	97.8
Others**	9	2.2
**Education Level**		
No formal education	38	9.1
Primary education	105	25.2
Secondary education	125	30.0
College and above	149	35.7
**Occupation**		
House Wife	228	54.7
Merchant	25	6.0
Government employee	91	21.8
Self-employee	40	9.6
Student	33	7.9
**Monthly income (ETB)** ***		
<600	72	17.3
601–1650	83	19.9
> = 1651	262	62.8
**Place of residence**		
Urban	396	95.0
Rural	21	5.0

* Tigre, Guraghe

**Never married, Widowed, Divorced, ETB- Ethiopian Birr

*** Ethiopia’s taxing classification was used

### Maternal and child health service utilization

Majority, 319 (76.5%) of the respondents had less than three live births and about, 388 (93.04%) of participants have attended antenatal care (ANC) for their previous or last pregnancy. Almost three fourth 300 (75.4%) of the respondents have started ANC visit at less than or equal to 4 months of gestational age. While only 68(16.3%) of the respondents came for postnatal check-up within fourteen days, the majority (83.7%) visited post natal care after fourteen days. About 107 (25.7%) of the respondents had been visited by health extension worker in the last six weeks and of which, 101(94.4%) of the respondents had been educated on early initiation of breastfeeding while above three-fourth, 329 (79.9%) of women have been advised on early initiation of breastfeeding, by skilled birth attendants before and after birth (Table 2).

**Table 2:** Obstetric characteristics of respondents, Nekemte city, Western Ethiopia, 2017.

Obstetric characteristics	Frequency	Percentage
Number of alive children		
1–2	319	76.5
3–4	74	17.7
5 and above	24	5.8
**ANC follow up**		
Yes	388	93.04
No	29	6.96
**Frequency of ANC**		
Once	7	1.8
Twice	37	9.5
Three times	83	21.4
Four times	261	67.3
**Time of PNC**		
Within 14 days	68	16.3
After 14 days	349	83.7
**Home visit by HEW**		
Yes	107	25.7
No	310	74.3
**Counselling on BF by HEW**		
Yes	101	94.4
No	6	5.6

BF- Breast feeding, HEW- Health extension workers, PNC- Postnatal care

### Essential new-born care practices

About 97(23.3%) of the respondents were reported as they had applied something on the cord, among these 81(83.5%) and 16 (16.5%) of women had applied butter and Vaseline respectively. Most of the respondents 325 (77.9%) have reported as they would to go health centre if there was cord infection and 79(18.9%) of women would give home medication, while 65(15.6%) of them reported as they would wait until it heals by itself. About 114 (27.3%) of women took care of the cord not to bleed while 313(75.1%) of women kept the cord dry and clean.

**Fig 1:**
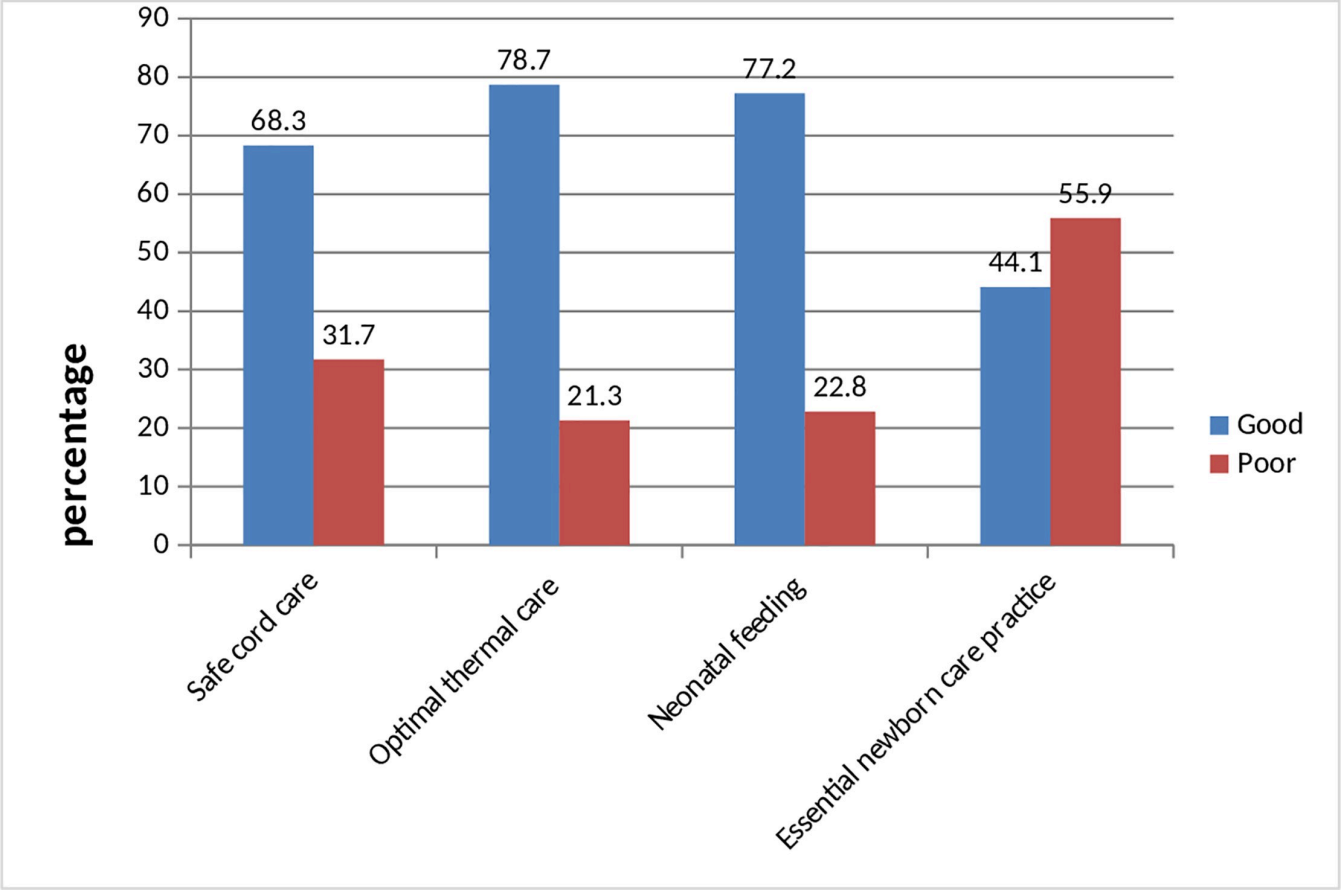
A percentage showing essential new-born care practices and its components among postnatal mothers, Nekemte city, Western Ethiopia, 2017.

The overall safe cord care practice of the respondents was 285 (68.3%) while the optimal thermal care practices and good neonatal feeding were 328 (78.7%) and 322 (77.2%) respectively. However, only 184 (44.1%) of the respondents practice Essential new-born care Fig 1.

Nearly half of the respondents 208 (49.9%) have good knowledge of essential new-born care practices. Mothers’ knowledge of new-born danger signs was low as only 153 (36.7%) of respondents were knowledgeable.

### Factors associated with essential new-born care practices

In this study, access to maternal health services, knowledge of essential new-born care, and counselling about essential new-born were factors significantly associated with essential new-born care practices.

Mothers who attended antenatal care services were found to have a statistically significant association with the practice of essential new-born care practices. Those who attended ANC visit at least once were 4.38 times more likely to practice essential new-born care practices as compared with those women who didn’t visit ANC at all for the current delivery [AOR = 4.38, 95% CI = (1.38, 13.94)]. Mothers who got counselling on essential new-born care by skilled birth attendant or health extension workers were more likely to have good essential new-born care practices compared to their counterparts [AOR = 2.32, 95% CI = (1.38, 3.91)]. Knowledge of mothers about essential new-born care was also another factor which had positive association with essential new-born care practices. Those who had good knowledge were 4.58 times more likely to practice essential new-born care [AOR = 4.58, 95% CI = (2.93, 7.16)] (Table 3).

**Table 3:** Factors associated with essential new-born care practices, Nekemte city, Western Ethiopia 2017.

Characteristics	Good practice	Poor practice	COR 95%CI	P-value	AOR 95%CI	P-value
**Residence**						
Urban	178(42.7)	218(52.3)	2.04(0.78, 5.40)	0.148	1.31(0.43, 3.94)	0.633
Rural	6(1.4)	15(3.6)	1.00		1.00	
**Place of delivery**						
Home	7(1.7)	15(3.6)	0.58(0.23,1.44)	0.188	1.25 (0.42, 3.71)	0.686
Health institution	177(42.4)	218(52.3)	1.00		1.00	
**Knowledge of neonatal danger sign**						
Yes	80(19.2)	73(17.5)	1.69(1.13,2.52)	0.011	0.99(0.62, 1.57)	0.948
No	104(24.9)	160(38.4)	1.00		1.00	
**ANC follow up**						
Yes	180(43.2)	208(49.9)	5.41(1.85, 15.83)	0.002	*4*.*38 (1*.*38*, *13*.94)*	0.012
No	4(0.95)	25(6.0)			1.00	
**Knowledge of essential new-born care**						
Yes	130(31.2)	78(18.7)	4.78(3.15, 7.27)	<0.0001	*4*.*58 (2*.*93*, *7*.16)*	<0.0001
No	54(12.9)	155(37.2)			1.00	
**Got counselling about new-born care**						
Yes	153(12.7)	145(34.8)	2.99(1.88, 4.78)	<0.0001	*2*.*32 (1*.*38*, *3*.91)*	0.002
No	31(7.4)	88(21.1)			1.00	
**Time of postnatal care check-up**	24 (5.8)	12(2.9)	0.65(0.21, 1.34)	0.241	0.57 (0.26, 1.26)	0.162
Within 6 days						
7–14 days	12(2.9)	20(4.8)	2.16(1.02, 4.56)	0.043	1.75 (0.78, 3.94)	0.178
After 14 days	197(47.2)	152(36.5)	1.00		1.00	

Where, AOR = Adjusted odd ratio, COR = Crude odd ratio, CI = confidence interval

*shows significant association at P<0.05

## Discussion

The current study identified magnitude of essential new-born care practices and factors affecting it in Nekemte Public health institutions. This study has found out that 68.3% of women have reported safe cord care practice. This result is higher than study conducted in Nepal (60%), Cameroon (11%), Bangladesh (42.8%) and the survey conducted in four regions of Ethiopia (65.2%) [11,15,21,22]. The possible reason for the variation might be socio economic differences between countries and difference in time of studies. On the other hand this finding was lower than the study conducted in Chitwan district (95%), and study done in East Gojjam (94.6%) [14, 23].

In this study about 23.3% of the women have applied different traditional substances on the cord such as butter and Vaseline. Even though the proportion varies, similar studies were being reported in Uganda (50%), Nepal (40%), Bangladesh (43.8%), Cameroon (54.2%), and the study done in India (55%) [11,12,21,22,24]. The possible reason that mothers apply a substance on the cord might be because; many people think that applying butter or Vaseline would lubricate the cord and prevent dryness [25,26].

The practice of early initiation of breastfeeding in the current study was 77.2%. This is almost similar to a finding of 2016 Ethiopian Demographic and Health survey (EDHS) report (73%) [3]. But, the current finding is higher than study done in Uganda (57%), Cameroon (44.3%), Chitwan district (24.3%), the survey conducted in four regions of Ethiopia (52.1%), and study conducted in East Gojjam, Ethiopia (41.6%) [10–12, 14, 15, 23]. In this study, about 94.4% of respondents were counselled about breastfeeding by health extension workers and delivery at health institution was high. This might have resulted in better proportion of early initiation of breastfeeding. However; this finding is lower than the study done in Nepal (90%) [21]. This shows that much effort is still needed to improve recommended breast feeding practice in the country. The current study has also showed that 97.4% of mothers had given colostrum, 77.2% initiated breastfeeding early, and 12.23% of them gave additional fluid for their current new-born before six months. This figure is better than the national EDHS report [3] may be because most (95%) of the respondents of this study were from urban and accessible to institutional delivery.

Optimum thermal care is another component of essential new-born care practices. In the current study 78.7% of women provided optimal thermal care for their new born. They provided it by delaying bathing of the new born 24 hours and wrapping the baby with clean clothes. This finding is higher than study done in Chencha district, Southern Ethiopia (71.0%), Nepal (60%), Uganda (42%), Cameroon (70.3%), India (64%), the survey conducted in 4 regions of Ethiopia (25.3%), and the study done in East Gojjam (34.4%) [11,12,14,15,24,26,27]. The difference may be due to Ethiopia’s health extension program which has improved women’s antenatal and postnatal care utilizations [28]. However, the finding of the study was found to be lower than the study done in the Chitwan district (96.7%) and Dessie, Northern Ethiopia (80.8%) [16, 23].

Overall, the level of essential new-born care practice was 44.1%. This is nearly similar with a study conducted in Chencha district of Ethiopia (38.4%) and Dessie town (46.9%) [16, 26]. However, this finding was higher than studies conducted in Northern parts of Ethiopia like Aksum (26.7%), Bahirdar (13.8%), and Damot Palusa (24%) [17–19]; but lower than study conducted in Mekelle, Ethiopia (81.1%) [20]. This may be as a result of variation of respondents; as those studies conducted in Bahir Dar and Damot Palusa were community based while this study was institution based.

Visiting ANC, access to counselling on essential new-born care and knowledge of the mothers about essential new-born care practices were independent predictors of essential new-born care practices. Those women who had access to ANC visit were 4.38 times more likely to practice essential new-born care as compared with those women who didn’t visit ANC at all for the current delivery. This finding is consistent with studies conducted in Bahirdar, Damot Palusa, and Dessie [11,14,15]. The possible reason could be those mothers who visited ANC would get counselling on advantages of delivery by skilled birth attendants, and institutional delivery which believed to increase knowledge and practice of mothers about essential new-born care [29].

The overall knowledge of mothers about essential new-born care practices was also a positive predictor of essential new-born care practices. Those who had good knowledge of ENBC were 4.58 times more likely to practice essential new-born care. This finding is consistent with a study done in Nepal, Chitwan district and Mekelle [20, 21, 23] which reported similar findings. This is due to the fact that accumulated experience and awareness about safe cord care, thermal care and breastfeeding would help mothers to put essential new-born care in to practice [20].

The present study found out that mothers who got counselling on essential new-born care by skilled birth attendant or health extension workers were more likely to have good essential new-born care practices compared to their counterparts. Studies conducted in Northern parts of Ethiopia; Aksum, Mekelle, Damot Palusa, and East Gojjam [14,17,19,20] similarly revealed that counselling during pregnancy, child birth and post natal period has positive association with essential new-born care practices after delivery. A similar study conducted in Goba town, Ethiopia also revealed that counselling is a positive predictor of ENBC practices [30]. This may be because access to counselling services would enhance mothers’ awareness and practice of essential new-born care [28].

### Strengths

The study covers wide area of essential new-born care components which are basically practiced by mothers at home such as: Neonatal feeding, safe cord care and thermal care. Even though it is facility based study, the study finding can be generalized to the target population due to the fact that currently, almost all mothers who have less than one year child attend immunization service for their infant.

### Limitations

There were some limitations to this study. This study is at a risk of social desirability bias as mothers may not report what they really practiced. In addition since data collection time was extended period, the study is not without recall bias as well.

## Conclusion

The present study indicated that the level of essential new-born care practice is unsatisfactory even though a majority of the respondents practice individual components of new-born care (Neonatal feeding, safe cord care and thermal care). Having ANC visit, knowledge of mothers about ENBC practices and getting counselled about care of the new-born were found to be independent predictors of essential new-born care practices. Promotion of essential new-born care through the provision of community-oriented awareness creation forum and counselling and promotion of antenatal care follow up education on essential new-born care and neonatal danger signs to all pregnant women are very important.

## Supporting information

S1 Dataset (SAV)
